# Implementation of a low-cost Interim 21CFR11 compliance solution for laboratory environments

**DOI:** 10.1155/S1463924603000129

**Published:** 2003

**Authors:** Jack E. Greene

**Affiliations:** Alkermes Inc. 88 Sidney Street Cambridge MA 02139 USA

## Abstract

In the recent past, compliance with 21CFR11 has become a major buzzword within the pharmaceutical and biotechnology industries. While commercial solutions exist, implementation and validation are expensive and cumbersome. Frequent implementation of new features via point releases further complicates purchasing decisions by making it difficult to weigh the risk of non-compliance against the costs of too frequent upgrades. This presentation discusses a low-cost interim solution to the problem. While this solution does not address 100% of the issues raised by 21CFR11, it does implement and validate: (1) computer system security; (2) backup and restore ability on the electronic records store; and (3) an automated audit trail mechanism that captures the date, time and user identification whenever electronic records are created, modified or deleted. When coupled with enhanced procedural controls, this solution provides an acceptable level of compliance at extremely low cost.


Journal of Automated Methods & Management in Chemistry
Vol. 25, No. 3 (May–June 2003) pp. 69–71

Taylor & Francis

Taylor & Francis Group

### Implementation of a low-cost Interim 21CFR11 compliance solution for laboratory environments†

**Jack E. Greene***
*Alkermes, Inc., 88 Sidney Street, Cambridge, MA 02139, USA*

*In the recent past, compliance with 21CFR11 has become a major buzzword within the pharmaceutical and biotechnology industries. While commercial solutions exist, implementation and validation are expensive and cumbersome. Frequent implementation of new features via point releases further complicates purchasing decisions by making it difficult to weigh the risk of non-compliance against the costs of too frequent upgrades. This presentation discusses a low-cost interim solution to the problem. While this solution does not address 100% of the issues raised by 21CFR11, it does implement and validate: (1) computer system security; (2) backup and restore ability on the electronic records store; and (3) an automated audit trail mechanism that captures the date, time and user identification whenever electronic records are created, modified or deleted. When coupled with enhanced procedural controls, this solution provides an acceptable level of compliance at extremely low cost.*

### Introduction

In 1997, the FDA enacted 21CFR11. This rule introduced additional requirements surrounding the use and storage of electronic records and electronic signatures for all systems regulated by the US Federal Food, Drug, and Cosmetic Act and the Public Health Service Act.

The electronic records portion of this act can be interpreted in light of the way that traditional paper records were maintained before the use of electronic systems became ubiquitous.

### Historical comparison

*How laboratory raw data was collected in 1980*

The most common form of laboratory data collection was onto paper records. Data was handwritten onto sequentially numbered worksheets/notebook pages or were written by instruments onto chart recorders. Instruments were set up and run by adjusting dials or keypads on the front panels.

*How laboratory raw data was managed in 1980*

Analysts wrote in notebooks with numbered, sewn pages or on sequentially numbered, logged worksheets and were trained to sign and date their work directly. Data deletions were obvious because a numbered page would need to be removed and destroyed, which would be obvious in any audit. On occasion where data was incorrect and required changing, analysts lined out the data such that the original data was not obscured. They then initialled the line out with a date, initials and justification for change.

*How laboratory raw data is collected today*

With the advent of high-speed computer hardware and software, laboratory systems have changed. Most equipment is run from a PC interface, and in many cases, operation without the PC is not possible. Data is collected onto the PC hard drive and processed and manipulated using software packages. In cases where these systems are connected to networks, data may be saved onto servers or LIMS systems.

*How laboratory raw data is managed today*

Analyst raw data is saved directly to hard drives. If data is to be signed and dated, they need to print out a hardcopy. Data deletions are difficult to detect because PC hard drives are difficult to audit. Data can be manipulated in both raw and processed form multiple times. In most cases, there is no way to detect this activity from the printout. Data can be copied and circulated such that it is difficult to determine which copy is authoritative.

The FDA is trying to maintain the same level of control over electronic records as was the case for paper records.

### Issues

There are some physical and logical security issues to solve such as limiting access to laboratory instruments as well as to laboratory raw data.

There are audit trail issues to solve centering around a mechanism to track when data is created, altered, deleted, by whom, and on what date and time.

There are revision control issues to solve. If data becomes modified, how can previous versions be retrieved?

### Commercial solutions

Most of the common solutions of these issues involve the use of a database. If data resides in databases, these

* e-mail: jack.greene@alkermes.com
†This paper was initially presented at the ISLAR 2002 Conference and is reproduced here by kind permission of Zymark Corporation.

systems can be used to manage version control as well as to provide audit trails. Users can log into databases that can enforce access rights that prevent deletions or modifications.

The core issues with all these systems centres around cost and flexibility. LIMS systems are expensive to purchase, difficult to configure and frequently limit how users use their systems. The validation effort on these systems is an entire separate undertaking with large amounts of work and associated costs.

### Alternative solutions

Networked operating systems can be used to provide a good level of physical and logical data security. If this is coupled with limited physical access and procedural controls, the overall solution can meet the needs of 21CFR11 including:

- Centralized access control list that applies to all computers on the laboratory network.
- Centralized password policy and account lockout.
- Prevent unauthorized users from gaining access.
- Allow for network-based data store.
- User groups by functional area with login restriction.
- Minimum password length and uniqueness.
- Password ageing, minimum and maximum password change times.
- Account lockout for too many failed login attempts.

While this schema solves the security issues relatively well, is has serious issues in the area of version control. If the operating system is the user interface to the instrument and its raw data, there is a rule of thumb: if users have the permission to save data, they also have the permission to modify or delete data. While this is not absolute, it holds in most cases.

This issue can be tackled using some basic IT tools. If data is not located on the local computer hard drive, then it is easier to control and manage. Laboratory users can be set to map secure areas of fileservers that have backup/restore systems. Laboratory software packages can be configured (automatically or procedurally) to save data directly to this secure network area.

This secure area can have Windows NT® Security Auditing set-up (assuming that this is your server operating systems) to capture the date, time and login ID whenever files are created, modified, accessed or deleted within the secure area. If this audit trail is examined, it can tell when a file is deleted, which allows for retrieval from backup tape.

### Issues to solve

While this schema does provide an audit tail and does allow for the retrieval of modified or deleted data, the main issue is how to read the audit trail. Due to the low-level nature of the Windows NT security log, the entries are very lengthy and difficult to interpret. The creation of a single file and its deletion occupies eight pages of log. Here is the final page of the log for such an action:

```
6/20/2002, 1:13:44 PM, Security, Success Audit, Object Access, 562, NT
AUTHORITY\SYSTEM, DATASERVER, Handle Closed:
   Object Server:   Security
   Handle ID:       2408
   Process ID:      2163827264

6/20/2002, 1:13:44 PM, Security, Success Audit, Object Access, 564, NT
AUTHORITY\SYSTEM, DATASERVER, Object Deleted:
   Object Server:   Security
   Handle ID:       2408
   Process ID:      2163827264

6/20/2002,1:13:44 PM, Security, Success Audit, Object Access, 560, S-1-5-21-823792125-1069754216-1537874043-1103, DATASERVER, Object Open:
   Object Server:        Security
   Object Type:          File
   Object Name:          E:\Data\RawQC\QC-
                         WKST-01 \script5.#01
   New Handle ID:        2408
   Operation ID:         {0,507671965}
   Process ID:           2163827264
   Primary User Name:    SYSTEM
   Primary Domain:       NT AUTHORITY
   Primary Logon ID:     (0x0,0x3E7)
   Client User Name:     user_test
   Client Domain:        LAB-DOMAIN
   Client Logon ID:      (0x0,0x1E426FD5)
   Accesses              DELETE
   Privileges            –

6/20/2002,1:13:44 PM, Security, Success Audit, Object Access, 562, NT
AUTHORITY\SYSTEM, DATASERVER, Handle Closed:
   Object Server:   Security
   Handle ID:       1380
   Process ID:      2163827264
```

While all of the critical information is in the log, an average day's log runs 20 000–30 000 pages and is not human readable.

### Solutions

The solution to the impenetrable log is to write a filtering program to parse the log into human-readable format. The ideal language for filtering large text files is perl (practical extraction and report language). Once a perl filter script is written, it can read the comma delimited Windows NT Security log, filter the log and output into Excel. This filter can then be validated to discover what a file creation and a file deletion looks like in the log for each instrument software package.

These tools need to be backed with audit trail procedural controls that require the following:

- Frequent backup of the secure raw data store.
- Collection and processing of the log.
- Review of the filtered log for the presence of overwrites or deletions before backup tapes are recycled.
- If overwrites or deletions are detected, restoration of files from tape and investigation into the occurrence.

There also need to be instrument procedural controls that mandate the following:

- User logins are user specific and that use of another user's login is prohibited.
- Modification or deletion of raw data is prohibited.
- Data systems have audit trails in place that track user identification, date and time.
- Use of password-protected screen savers is required.
- Initiation of data collection during network failure is prohibited (no audit trail running).
- What to do if network failure occurs during data collection (depends on the software package).

### Summary

There are still the following issues to address with the system:

- If a file is created and then modified/deleted before the server is backed up, then that version is lost (the log will capture the creation and the modification/deletion event, but the data will be lost).
- Filter development and validation is an iterative process that is time consuming.
- Investigations are difficult and frequently have false-positives as the root cause.
- Deployment and management of the system require agreement and cooperation of IT, QC and Validation to be successful.

While this alternative solution is far from perfect, it has the strong benefit of using standard IT tools (servers, networks and operating systems) that are understood and usually already exist in-house. The software development effort to write the filter script is small and the resulting code is short and straightforward to explain to inspectors. When coupled with enhanced procedural controls, this solution provides an acceptable level of compliance at extremely low cost.

